# Nuclear Magnetic Resonance Based Profiling of Biofluids Reveals Metabolic Dysregulation in HIV-Infected Persons and Those on Anti-Retroviral Therapy

**DOI:** 10.1371/journal.pone.0064298

**Published:** 2013-05-16

**Authors:** Saif Ullah Munshi, Bharat Bhushan Rewari, Neel Sarovar Bhavesh, Shahid Jameel

**Affiliations:** 1 Virology Group, International Centre for Genetic Engineering and Biotechnology, New Delhi, India; 2 National AIDS Control Organization, New Delhi, India; 3 Structural and Computational Biology Group, International Centre for Genetic Engineering and Biotechnology, New Delhi, India; National Research Council of Italy, Italy

## Abstract

**Background:**

Although HIV causes immune deficiency by infection and depletion of immunocytes, metabolic alterations with clinical manifestations are also reported in HIV/AIDS patients. Here we aimed to profile metabolite changes in the plasma, urine, and saliva of HIV/AIDS patients, including those on anti-retroviral therapy (ART).

**Methods:**

Metabolic profiling of biofluids collected from treatment naïve HIV/AIDS patients and those receiving ART was done with solution-state nuclear magnetic resonance (NMR) spectroscopy followed by statistical analysis and annotation.

**Results:**

In Principal Component Analysis (PCA) of the NMR spectra, Principal Component 1 (PC1) alone accounted for 99.3%, 87.2% and 78.8% variations in plasma, urine, and saliva, respectively. Partial least squares discriminant analysis (PLS-DA) was applied to generate three-component models, which showed plasma and urine to be better than saliva in discriminating between patients and healthy controls, and between ART-naïve patients and those receiving therapy. Twenty-six metabolites were differentially altered in any or two types of samples. Our results suggest that urinary Neopterin, and plasma Choline and Sarcosine could be used as metabolic biomarkers of HIV/AIDS infection. Pathway analysis revealed significant alternations in 12 metabolic pathways.

**Conclusions:**

This study catalogs differentially regulated metabolites in biofluids, which helped classify subjects as healthy controls, HIV/AIDS patients, and those on ART. It also underscores the importance of further studying the consequences of HIV infection on host metabolism and its implications for pathogenesis.

## Introduction

The human immunodeficiency virus (HIV) primarily infects CD4+ cells, especially T cells and monocytes. Though these cells have no major role in host metabolism, clinical features of end-stage HIV/AIDS patients show gross metabolic changes, manifested as weight loss and malnutrition [1], the main reasons for which are malabsorption, increased energy expenditure and metabolic alterations [2]. Additional factors that might contribute to this are endocrine dysfunction and the metabolic cost of inflammation, including cytokine production [3]. Abnormalities in energy, protein, lipid and glucose metabolism have been reported in HIV/AIDS patients since recognition of the disease and introduction of ART. Following infection, several factors including HIV itself, opportunistic infections (OIs), the host immune response and ART modulate metabolic changes either directly or indirectly [4]. Hypermetabolism was reported in the early asymptomatic phases of infection [5], [6] and the severity of the resting hypermetabolic response appears to increase in all the stages of the disease, especially due to secondary infections [6], [7]. Besides immunodeficiency, HIV infection and its treatment cause metabolic derangement and associated clinical features. Thus, understanding metabolic changes during different stages of HIV infection is critical for proper patient management.

Due to its intimate contact with tissues, blood is the most representative of all biofluids. Blood plasma is filtered through the kidneys to produce urine, which contains waste products, including metabolites. Homeostasis in the oral cavity is maintained by normal oral flora and saliva, which has immunomodulatory, anti-inflammatory and antimicrobial activities against different pathogens. Therefore, a comprehensive analysis of the patient plasma, urine and saliva metabolomes is important for identifying metabolic changes in HIV/AIDS patients. Metabonomics aims to understand the metabolic composition of biological fluids and tissues [8], and is performed through profiling with nuclear magnetic resonance (NMR) or mass spectrometry (MS), followed by statistical analysis of the data. A distinctive metabolic fingerprint is generated for each disease sample, which is then compared with healthy samples. These methods have been used to study coronary heart disease [9], Alzheimer's disease [10], viral hepatitis caused by hepatitis B, C or E viruses [11]–[13] and bacterial meningitis [14]. Metabolic profiling was also used to segregate sera [15], [16] or saliva [17] of different groups of HIV patients using NMR and MS, respectively. In this study, we have elucidated HIV-mediated effects on plasma, urine and saliva metabolites and changes in patients who are on ART. This pilot study further demonstrates the feasibility of applying metabonomics to identify new metabolic biomarkers and correlate these changes to clinical features observed in HIV/AIDS patients. We found 26 metabolites to be differentially regulated either in one or two of the biofluids. Based on this, we propose several metabolic pathways to be dysregulated in HIV/AIDS patients and those on ART.

## Methods

### Clinical samples

The study subjects were divided into two groups - HIV/AIDS patients who were ART naïve and those who were on ART for at least 6 months. The number of samples collected and their characteristics are shown in Table 1. The samples were collected from those attending the ART Clinic at the Dr. Ram Manohar Lohia (RML) Hospital in New Delhi, India. Those on ART received a combination of Zidovudine (ZDV), Lamivudine (3TC) and Nevirapine (NVP)/Efavirenz (EFV), or Stavudine (d4T), 3TC and NVP/EFV. The healthy controls were HIV-negative. The study subjects had no known immunological manifestations, metabolic conditions or co-infections (tuberculosis, viral hepatitis B or C), and were not receiving any anti-tubercular drugs. Five ml of blood was collected in an EDTA-vacutainer, allowed to stand for 30 min at room temperature and kept on ice before separating the plasma. Random urine samples (150–200 ml) were collected in 250 ml sterile bottles, kept on ice and centrifuged at 1000 xg for 10 min. To avoid metabolite variations in urine, it was ensured that the study subjects had not taken any food or drink at least two hours before sampling. For collection of saliva, the subjects were advised to rinse their mouth thoroughly with deionized water and to rest for 5 min before saliva was allowed to drip off the lower lip into a sterile tube. Whole, unstimulated saliva (1–3 ml) was collected over a 5 min period, kept on ice, centrifuged at 12,000 xg for 10 min to remove cells, and the supernatants were collected. The plasma, urine and saliva samples were divided into aliquots and stored at −70°C.

**Table 1:** Details of clinical samples.

Group	Number (Male:Female)	Mean age; years (range)	Mean CD4 counts/ µl (range)
HIV/AIDS; ART naïve	Blood 23 (16∶7)	32 (15–48)	249 (14–637)
	Urine 27 (20∶7)	32 (15–51)	226 (14–632)
	Saliva 21 (14∶7)	33 (15–48)	244 (14–632)
HIV/AIDS; ART+	Blood 14 (11∶3)	36 (24–51)	466 (282–698)
	Urine 18 (15∶3)	32 (24–51)	486 (282–736)
	Saliva 12 (10∶2)	34 (24–51)	510 (316–736)
Healthy controls	Blood 12 (9∶3)	32 (25–48)	ND
	Urine 12 (9∶3)	32 (25–48)	ND
	Saliva 8 (7∶1)	34 (25–48)	ND

### Ethics statement

Institutional Review Boards of the National AIDS Control Organization (NACO), New Delhi, India and Ram Manohar Lohia Hospital, New Delhi, India, approved this study. Potential study subjects were selected based on their case records and the study was explained to them by one of the investigators in the presence of a social worker. For subjects under 18 years of age, the study was also explained to an accompanying relative or guardian. Only those subjects that either gave informed written consent themselves, or whose accompanying relative/guardian provided it, were included in the study.

### NMR spectroscopy

All NMR experiments were carried out at 25°C on a Bruker Avance III spectrometer equipped with cryogenic triple-resonance TCI probe head, operating at the field strength of 500.13 MHz. Temperature calibration was performed using a 100% d_4_-methanol sample. The NMR spectra were processed using Topspin 2.1 (Bruker Corporation). Samples were prepared using 100 µl of plasma, 400 µl of urine or 100 µl of saliva; the volume was made up to 500 µl with D_2_O (Deuterium oxide, ^2^H>99.8%) and 0.0005% (*w/v*) DSS. These were placed in 5 mm NMR tubes (Wilmad, Sigma Aldrich, USA). One-dimensional ^1^H spectra of urine and saliva were measured using 1D gradient enhanced nuclear Overhauser enhancement spectroscopy (NOESY)-presaturation (mixing time 10 ms) [18]. For plasma, the Carr–Purcell–Meiboom–Gill (CPMG) echo sequence was used to partially suppress the protein signals [19]. Based on the experimental optimization, a total CPMG delay of 300 ms was used with an echo time of 200 µs. All one-dimensional ^1^H NMR spectra were measured with 32 scans with a relaxation delay of 4 sec. For each sample free induction decays (FIDs) were collected with a spectral width of 10,000 Hz and acquisition time of 3.28 sec (*t_1max_*). The plasma, urine and saliva spectra were referenced to DSS at 0 ppm. One-dimensional ^1^H spectra of a few samples were measured in duplicate with an interval of 30 min to explore the stability of metabolites at 25°C. The spectral region between 6.0 and 4.5 ppm was set to zero integral to remove the effects of suppression of water resonance and variation in the urea signal caused by partial cross-solvent saturation due to solvent-exchanging protons. In the plasma spectra single NMR peaks close to the EDTA signal were not used for quantitation. Since the levels of plasma and urinary Creatinine vary significantly among HIV/AIDS patients and those on ART [20], standardization of urine samples using Creatinine was avoided. Instead, we used the normalization to a constant sum method, which is a commonly used metabolomic normalization method, and is part of MetaboAnalyst [21]. Two-dimensional phase sensitive gradient enhanced echo-antiecho [^13^C,^1^H]-HSQC spectra were measured on saliva, plasma and urine samples [22]. The spectrum was acquired with 160 scans per t_1_ increment in ^13^C dimension. Acquisition times for ^13^C and ^1^H dimension were 7.95 ms (*t_1max_*) and 127.85 ms (*t_2max_*) respectively. Spectral-widths for ^13^C and ^1^H dimensions were 20122.86 Hz and 8012.82 Hz, respectively. Carrier frequencies for ^13^C and ^1^H dimensions were kept at 80 ppm and 4.7 ppm, respectively.

### Statistical analysis, metabolite identification and pathway analysis

Statistical analyses and annotations of the NMR spectra were carried out using MetaboAnalyst [21]. This web-based program contains several statistical and machine learning algorithms, including chemometric analysis. The intensities of collected NMR spectra were converted to.cvs files, which were uploaded in MetaboAnalyst. Through different statistical analyses, a list of NMR peaks with their relative quantity, which are responsible for separation of different study groups, was identified. The corresponding metabolites for those peaks were recognized from the Human Metabolome Data Bank (HMDB) [23] and MetaboMiner [24], which are linked with MetaboAnalyst. We confirmed the list of metabolites by comparing their ^1^H and ^13^C chemical shifts and *J*-coupling patterns of resonances with previously published values [25], [26] or those available from HMDB. We also reconfirmed metabolites using data acquired from 2D [^13^C,^1^H]-HSQC spectra through Metabominer [24]. Metabolic pathway analysis was carried out using MetPA (http://metpa.metabolomics.ca) [27].

## Results

### Multivariate analysis of metabonomics data

**Figure 1:**
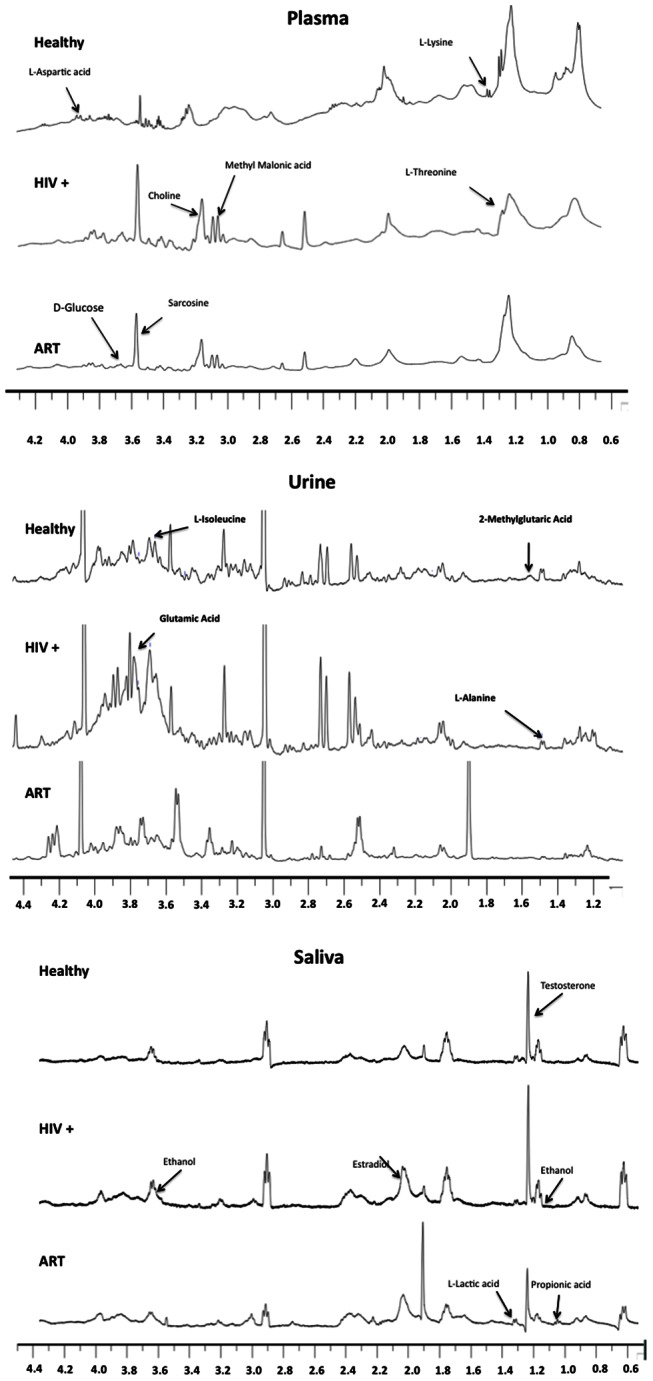
Representative one-dimensional ^1^H NMR spectra of plasma, urine, and saliva obtained from a healthy control, a HIV/AIDS patient, and a patient on ART. The differences in the intensity of metabolites in different samples are indicated with arrows.

**Figure 2:**
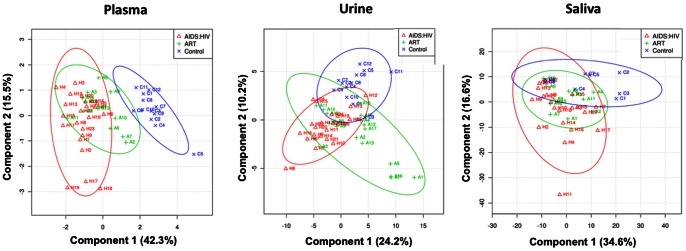
Parital least squares discrimination assay (PLS-DA) two-dimensional score plots developed from the ^1^H NMR spectra of plasma, urine, and saliva collected from healthy control (X) and HIV/AIDS patients (Δ), and HIV/AIDS patients on ART (+).

One-dimensional ^1^H-NMR spectra of plasma, saliva and urine from patients and controls are presented in Figure 1. No metabolite degradations were observed in any sample when duplicate spectra were measured after an interval of 30 min. Since the spectra were complex and showed variations between individuals, we used chemometric methods such as Principal Component Analysis (PCA) and Partial Least Squares Discrimination Assay (PLS-DA). PCA was performed with the first three principal components (PCs) on the plasma, urine and saliva data sets to distinguish between patients and controls on the basis of their NMR spectra. In this analysis, PC1 alone explained 99.3%, 87.2% and 78.8% variations in plasma, urine and saliva respectively. To describe maximum separation between pre-defined sample-classes, the supervised PLS-DA method was used. Each sample was first classified into one of three groups – HIV/AIDS patients who were ART naïve, patients on ART and healthy controls; this was provided as a Y-table for the PLS-DA analysis. A regression analysis of the original data was performed against the Y-table. In the PLS-DA scores plot, the first three partial least squares (PLS) components - PLS1, PLS2 and PLS3 showed good separation between patients and controls (Fig. 2). To measure the robustness of the PLS-DA model, the fraction of variance explained by a model (*R^2^*) and the total variation predicted by the model (*Q^2^*), were used. A model with *R^2^*>0.7 and *Q^2^*>0.4 is regarded as good for biological data [28]. We obtained three-component models for plasma, urine and saliva with an accuracy of 0.81 (*R^2^* = 0.79, *Q^2^* = 0.70), 0.86 (*R^2^* = 0.80, *Q^2^* = 0.37) and 0.75 (*R^2^* = 0.63, *Q^2^* = −0.32), respectively. These results indicated that models generated from the changes in plasma and urine metabolites, but not those generated from saliva, to be good. The plasma and urinary metabolite models could accurately discriminate between the three groups of individuals.

### Identification of metabolites

**Figure 3:**
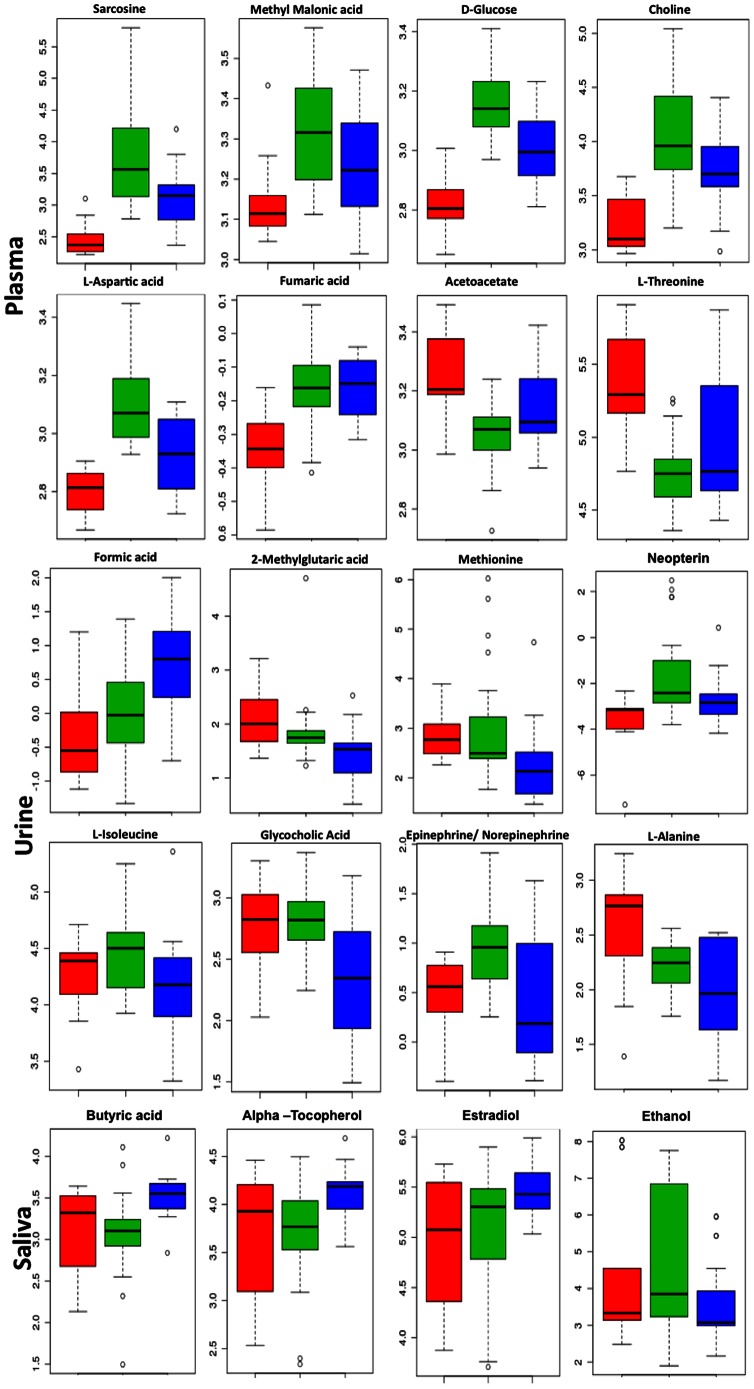
Box and whisker plots showing the levels of representative metabolites in plasma, urine, and saliva collected from healthy controls (red), HIV/AIDS patients (green), and patients on ART (blue). The y-axis shows the normalized concentrations of the metabolites.

In addition to classification, PLS-DA was used to select important features depending upon the variable importance in projection (VIP) score, which is a weighted sum of squares of the PLS loadings. Higher the VIP score in PLS-DA, higher is the importance of a variable, in the present case a metabolite or a group of metabolites. To identify the important features we set the VIP score at 0.8 [29]. In MetaboAnalyst, PLS-DA analysis also provided the patterns of change for those variables, i.e. concentration of the metabolites in arbitrary units in the plasma, urine and saliva data sets. The resonances corresponding to those variables are attributable to metabolites whose levels were significantly different either in plasma, urine or saliva of our study groups when compared with the control group (Table 2 and Fig. 3).

**Table 2:** Catalog of metabolites.

Metabolites	*ppm/multiplicity*	HIV	On ART
2-Methylglutaric acid/u	1.52/m	↓	↓↓
Acetoacetate/p	2.28/s	↓↓	↓
Alpha –Tocopherol/s	2.32/m	↓	↑
Butyric acid/s	2.16/m	↓	↑
Choline/p	3.2/s	↑↑	↑
D-Glucose/p	3.72/m	↑↑	↑
Epinephrine/u/Norepinephrine/s,u	6.94/m	↑	↓
Estradiol/s	2.04/br.s	↑	↓
Ethanol/s	1.16/t 3.64/q	↑	↑↑
Formic acid/u	8.44/s)	↑	↑↑
Fumaric Acid/p	8.52/s	↑	↑
Glutamic Acid/u	3.72/dd	↑	↑
Glycocholic Acid/u	2.08/m	–	↓
L-Isoleucine/u	3.66/d	↑	↓
L-Alanine/u	1.44/d	↓	↓↓
L-Aspartic acid/p	3.88/dd	↑↑	↑
L-Lactic acid/s	1.32/d	↑	↑
L-Lysine/p	1.4/m	↓	↓
L-Threonine/p	1.32/d	↓	–
Methionine/u	2.16/m	↑	↓↓
Methyl Malonic acid/p	3.12/q	↑↑	↑
Neopterin/u	8.68/s	↑↑	↑
Propionic acid/s	1.04/t	–	↑
Sarcosine/p	3.6/s	↑↑	↑
Sucrose/u	3.48/dd	↑	↑
Testosterone/s	1.2/s	↑	↓

The metabolites differentially regulated in plasma (p), urine (u) or saliva (s) in HIV/AIDS patients and patients on ART are shown. The arrows indicate lower (↓) or higher (↑) arbitrary levels of metabolites compared to healthy controls.

Compared to healthy controls, the levels of Sarcosine, Methylmalonic acid (MMA), D-Glucose, Choline and L-Aspartic acid were higher in the plasma of HIV/AIDS patients. Though the levels of these metabolites were reduced in patients receiving ART, they were still higher than those found in healthy persons. The levels of some other metabolites like 5β-Cholestanol, L-Lysine, Acetoacetate and L-Threonine were low in HIV/AIDS patients, increased in the ART group, but did not reach the levels found in healthy controls. In urine, Glutamic acid and Formic acid levels were higher in HIV/AIDS patients compared to healthy controls, and were even higher following ART administration. Alternatively, the levels of Neopterin and Epinephrine/Norepinephrine were higher in HIV/AIDS patients but lower concentrations were observed in patients receiving ART. The levels of Methionine, 2-Methylglutaric acid, L-Alanine and Glycolic acid were lower in the urine of HIV-infected persons; these were reduced further following ART. The metabolite changes in saliva were not significantly different between infected persons, those on ART and healthy controls. Only salivary L-Lactic acid levels were higher following infection and remained so on therapy. Alternatively, there was an elevation in the levels of Butyric acid, α-Tocopherol, L-Lysine and Propionic acid, and reduction in the levels of Testosterone and Ethanol in saliva following ART administration.

### Metabolic pathway analysis

The metabolites identified from our study were subjected to pathway analysis. We observed 12 metabolic pathways to be modulated significantly (*p*<0.05) in HIV infected patients (Table 3). These changes are especially prominent in metabolic cycles, glucose metabolism, hormone biosynthesis and amino acid biosynthesis pathways indicating that significant changes occur in metabolic processes during HIV infection and after the introduction of ART.

**Table 3:** Metabolic pathways modulated in HIV/AIDS patients.

Pathway Name	Total Compounds	Raw p value	Hits	Name of the Hits
Aminoacyl-tRNA biosynthesis	75	7.18E-05	7	L-Glutamine, L-Methionine, L-Lysine, L-Isoleucine, L-Threonine, L-Threonine, L-Aspartic acid
Glycine, serine and threonine metabolism	48	5.28E-04	4	Choline, Sarcosine, L-Threonine, L-Aspartic acid
Arginine and proline metabolism	77	0.0044999	4	L-Glutamine, L-Aspartic acid, Fumaric acid, Sarcosine
Propanoate metabolism	35	0.0014254	4	Methylmalonic acid, Propionic acid, L-Lactic acid, Acetoacetic acid
Nicotinate and nicotinamide metabolism	44	0.0033674	3	L-Aspartic acid, Fumaric acid, Propionic acid
Tyrosine metabolism	76	0.022891	3	Fumaric acid, Acetoacetic acid, Norepinephrine,
Alanine, aspartate and glutamate metabolism	24	0.004626	4	L-Glutamic Acid, Fumaric acid, L-Aspartic acid, L-Alanine
Glycolysis or Gluconeogenesis	31	0.0095828	3	D-Glucose, L-Lactic acid, Ethanol
Nitrogen metabolism	39	0.017998	3	L-Aspartic acid, L-Glutamic Acid, Formic Acid
Butanoate metabolism	40	0.019265	4	Butyric acid, Fumaric acid, Acetoacetic acid, L-Glutamic Acid
Starch and sucrose metabolism	50	0.034627	2	Sucrose, D-Glucose
Pyruvate metabolism	32	0.045640	2	L-Lactic acid, Formic Acid

The pathways that have at least 2 metabolites in the “hits” list and are differentially regulated in HIV/AIDS are shown.

## Discussion

In this pilot study, we have carried out metabolic profiling of plasma, urine and saliva collected from HIV/AIDS patients with and without ART, and healthy controls. The NMR spectra and metabolite resonance intensities were compared through multivariate statistical analyses, showing these to be separable. This indicated that the NMR signal intensity (i.e. concentration) of multiple metabolites varied between each group of samples and infection/disease-specific changes were observed in the biofluids. The changes were more significant for plasma and urine compared to saliva. Using a metabonomics approach, the metabolites differentially present in the biofluids were identified and these were extrapolated to pathway networks and systemic effects following HIV infection and ART administration.

### Abnormalities in amino acid levels

L-Aspartic acid, which is a non-essential amino acid synthesized from Glutamic acid and has important roles in the urea cycle and DNA metabolism, was elevated in HIV/AIDS patients and reduced following ART but not to the levels found in healthy individuals. In an earlier study, Citrulline levels were found to be significantly higher in HIV/AIDS patients [30]. We suggest that increased levels of L-Aspartic acid and Citrulline from the urea cycle may lead to elevated levels of plasma Fumaric acid in HIV/AIDS patients and those on ART observed in our study. In HIV/AIDS patients, the plasma levels of essential amino acids L-Lysine and L-Threonine were reduced, as observed previously [31]–[33]. Since these essential amino acids can only be obtained through diet, this indicates reduced dietary intake due to loss of appetite or malabsorption, both of which are frequently seen in HIV/AIDS patients. Elevated urinary L-Methionine indicates increased loss of this essential amino acid, resulting in low levels in the plasma of HIV/AIDS patients observed in earlier studies [31]–[33]. Methionine is rate limiting for whole-body protein synthesis in HIV/AIDS infection [32]. Lysine and Methionine synthesize Carnitine, which augments the production of Acetoacetate [34. As lack of total and free Carnitine was observed earlier in AIDS patients [35], we suggest that reduced Carnitine synthesis is probably due to reduced levels of Methionine, which results in lower levels of Acetoacetate observed in our patients. We also found elevated plasma levels of Sarcosine in HIV/AIDS patients, which reduced on ART, but was still higher compared to healthy controls Increased levels of Sarcosine were reported earlier in the oral metabolites of HIV/AIDS patients [17]. Sarcosine is converted to Glycine with the help of folate, which is deficient in HIV/AIDS patients [36]. This deficiency may lead to less conversion of Sarcosine to Glycine causing sarcosinemia in HIV/AIDS patients, which was observed in our study. Sarcosine is also a degradation product of muscles and other tissues, and its elevation may also be due to muscle wasting in HIV patients [37].

### Indicators of immune status in HIV patients

We found significant changes in two important indicators of immune function - Neopterin and Vitamin E. There was increased urinary excretion of Neopterin in HIV patients, which was reduced in the ART group. Neopterin is a catabolic product of guanosine triphosphate (GTP) synthesized by monocytes/macrophages, endothelial cells, B cells and dendritic cells, measurement of which in body fluids is indicative of a pro-inflammatory immune status [38]. There is strong inverse correlation between urinary and serum Neopterin and CD4+ lymphocyte counts in HIV infected persons [39]. Our findings confirmed earlier reports and showed a reversal of Neopterin increase following ART. Compared to healthy controls, we also found lower levels of salivary α-Tocopherol, a type of Vitamin E that acts as an antioxidant and immunostimulator, in HIV/AIDS patients; this increased in patients on ART. Increased urinary excretion of Vitamin E was suggested to be responsible for its reduced plasma levels in HIV patients [40]. We suggest that this may also be responsible for the reduced salivary Vitamin E, which was reversed on ART.

### Altered nervous system metabolites

Norepinephrine and Choline are precursors for Acetylcholine. We observed higher urinary and salivary Norepinephrine in HIV/AIDS patients, which reduced in patients on ART. In other studies, urinary Norepinephrine levels correlated positively with HIV viral load [41], and with depression and anxiety [42], which is frequently observed in HIV/AIDS patients. Norepinephrine also enhances the adhesion of HIV-1-infected leukocytes to endothelial cells of the cardiac microvasculature [43]. We found elevated levels of Choline in the plasma of HIV/AIDS patients, which was also observed previously [15]. In a study using ^1^H MRS imaging, increased Choline was detected in the subcortical area of the brain early in HIV disease, even when the infection was asymptomatic [44]. Choline is an important precursor for Acetylcholine and is required for membrane synthesis. A rise in Choline levels is associated with increased membrane turnover, demyelination or inflammation, and was observed in demyelinating diseases [45]. Following HIV infection, inflammatory demyelinating neuropathies are frequently diagnosed [46]. Increased levels of Choline, which are reduced on ART, indicate Choline to be a biomarker of HIV/AIDS, especially in the AIDS dementia complex [47].

### Dysregulation of sex hormones

HIV affects the ability to produce and maintain sex hormone levels in infected persons. We observed higher levels of salivary Testosterone in HIV/AIDS patients, which reduced on ART. Plasma levels of free, bioavailable and total Testosterone were previously reported to be low in HIV patients [47], and there was a positive correlation between serum Testosterone and CD4+ cell counts [48]. Though in the ART group we found decreased salivary Testosterone, HAART was earlier shown not to decrease its plasma levels [48]. This discrepancy between salivary and plasma Testosterone found in our study was noticed previously as well [49]. Additionally, as observed earlier [50] we found increased levels of salivary Estradiol in HIV/AIDS patients, which increased further on ART administration. The deregulation of sex hormones in HIV infection leads to diminished libido and impotence, which is often observed in HIV patients [51].

### Metabolic changes in the oral fluid

We found significant changes in three salivary metabolites - Butyric acid, Lactic acid and Ethanol, which may be associated with opportunistic pathogens prevalent in HIV/AIDS patients. Butyric acid is a product of *P. gingivalis* and *F. nucleatum*, which cause peridontal disease observed in these patients, including those on ART [52]. Butyric acid causes histone acetylation and can thus induce the reactivation of latent proviruses [53]. Our results indicate that even in patients on ART, despite improvements in general health, Butyric acid producing bacteria may remain in the oral cavity. Though the blood levels of Lactic acid are significantly higher in HIV/AIDS patients [54], we also found higher levels of Lactic acid in the saliva of patients, including those on ART. Instead of plasma lactic acidosis, this may be due to lactic acid producing bacteria in the oral cavity of HIV/AIDS patients [55]. We also found elevated levels of salivary Ethanol in the patients, which was reduced on ART. Oropharyngeal candidiasis caused by *C. albicans*, which produces Ethanol *via* anaerobic fermentation, is the most prevalent opportunistic infection in HIV/AIDS [56]. Since HIV infection of primary oral epithelial cells in culture is increased significantly by alcohol [57], we suggest that increased levels of Ethanol in the oral cavity may also increase the risk of continuous oral reinfection.

### Changes in other metabolites

In HIV/AIDS patients we observed increased plasma levels of Methylmalonic acid (MMA), a dicarboxylic acid that forms methylmalonyl-CoA, which is converted into succinyl-CoA in a vitamin B12-dependent process, and enters the Krebs cycle. Elevated levels of MMA are found in most patients with B12 deficiency, the latter also reported during HIV infection [58]. Thus, increased levels of MMA found in this study reaffirm Vitamin B12 deficiency of HIV/AIDS patients. Fumaric acid was also increased in plasma and 2-Methylglutaric acid was decreased in the urine of HIV patients and those on ART. Both of these are derived from Succinate and are metabolites of the Citric acid cycle, suggesting its deregulation during HIV infection.

### Metabolic pathway analysis

We observed differential production of metabolites in plasma, urine and saliva in response to HIV infection and their changes after the introduction of ART. Interestingly ART did not normalize increased levels of some metabolites such as plasma MMA, Sarcosine, Acetoacetate, urinary Neopterin, and salivary testosterone. There was a further increase with ART in the levels of some other metabolites such as urinary Formic acid, salivary Butyric acid and Ethanol, or a decrease in others such as urinary L-Alanine, Methionine and 2-Methylglutaric acid. Pathway analysis showed several metabolites in the amino acid biosynthesis, carbohydrate metabolism, steroid synthesis and nitrogen metabolism pathways to be differentially regulated between patients and controls. Among these metabolites, some such as D-Glucose, Sucrose, L-Glutamine, L-Aspartic acid and L-Glutamic acid are commonly involved in overlapping metabolic processes, and may correspond to principal regulatory points of the human metabolome in HIV infection. There are reports of dysregulation in Glucose [59] and amino acid metabolism [60] in HIV patients, and reducing plasma viral load improves muscle amino acid metabolism [61]. Undoubtedly these metabolic pathways are involved in general health and their dysregulation leads to gradual weight loss, muscle wasting, anorexia, malabsorption and malnutrition observed in HIV patients. The metabolites involved in amino acid metabolism may also play important roles in regulating host immunity [62], and changes in their levels impair immune function, increasing susceptibility to other infections.

## Conclusions

This study showed metabolites present in plasma, urine and to a lesser extent in saliva to be differentially produced in response to HIV infection, with significant changes in their levels following ART administration. Dysregulated metabolic pathways were also identified, which contribute to pathogenesis and disease outcome in HIV/AIDS patients.
